# A population-based study of the trend in SARS-CoV-2 diagnostic modalities from the beginning of the pandemic to the Omicron surge in Kyoto City, Kyoto, Japan

**DOI:** 10.1186/s12889-023-17498-3

**Published:** 2023-12-21

**Authors:** Hiroki Kyo, Shivani A. Patel, Masaki Yamamoto, Yasufumi Matsumura, Takeshi Ikeda, Miki Nagao

**Affiliations:** 1 MetroAtlanta Ambulance Service, Emory Healthcare Network, Atlanta, GA USA; 2 Hubert Department of Global Health, Rollins School of Public Health, Emory University, Atlanta, GA USA; 3 Department of Clinical Laboratory Medicine, Kyoto University Graduate School of Medicine, Kyoto, Japan; 4 Department of Clinical Laboratory, Kyoto University Hospital, Kyoto, Japan; 5 Public Health and Welfare Bureau of Kyoto City, Kyoto, Japan

**Keywords:** SARS-CoV-2, COVID-19, Testing modality, NAAT, Antigen testing, Active epidemiological investigation

## Abstract

**Background:**

The coronavirus disease 2019 (COVID-19) presents critical diagnostic challenges for managing the pandemic. We investigated the 30-month changes in COVID-19 testing modalities and functional testing sites from the early period of the pandemic to the most recent Omicron surge in 2022 in Kyoto City, Japan.

**Methods:**

This is a retrospective-observational study using a local anonymized population database that included patients' demographic and clinical information, testing methods and facilities from January 2020 to June 2022, a total of 30 months. We computed the distribution of symptomatic presentation, testing methods, and testing facilities among cases. Differences over time were tested using chi-square tests of independence.

**Results:**

During the study period, 133,115 confirmed COVID-19 cases were reported, of which 90.9% were symptomatic. Although nucleic acid amplification testing occupied 68.9% of all testing, the ratio of lateral flow devices (LFDs) rapidly increased in 2022. As the pandemic continued, the testing capability was shifted from COVID-19 designated facilities to general practitioners, who became the leading testing providers (57.3% of 99,945 tests in 2022).

**Conclusions:**

There was a dynamic shift in testing modality during the first 30 months of the pandemic in Kyoto City. General practitioners increased their role substantially as the use of LFDs spread dramatically in 2022. By comprehending and documenting the evolution of testing methods and testing locations, it is anticipated that this will contribute to the establishment of an even more efficient testing infrastructure for the next pandemic.

**Supplementary Information:**

The online version contains supplementary material available at 10.1186/s12889-023-17498-3.

## Introduction

The novel coronavirus disease 2019 (COVID-19), an emerging infectious disease, posed initial challenges in conducting widespread testing due to a shortage of testing kits and equipment from the early stages of the pandemic [1, 2]. On the other hand, coordinated surveillance systems that enabled the identification, contact tracing, and support for infected patients were essential for effective epidemic management [3, 4]. Due to the potential for SARS-CoV-2 to spread infection even from asymptomatic individuals, Japan conducted proactive epidemiological investigations and testing of close contacts as part of its efforts to control the pandemic. This led to an increased demand for the expansion of testing methods [5, 6].

In Japan, during the initial stages of the pandemic, the number of tests conducted was approximately 1/20th to 1/50th of that in Western countries [7]. However, as various testing kits were approved and began to circulate during the pandemic, the testing rate per 1000 people eventually reached a level similar to that of Western countries [7, 8]. This rapid transformation in the field of clinical testing was unprecedented before the COVID-19 era.

The aim of this study was to analyze the changes in the utilization of SARS-CoV-2 testing, including a shift in the test specimen samples during the early pandemic period to several peaks of infection from January 2020 to June 2022. The results can help us understand how the utilization of various diagnostic modalities during the COVID-19 response influenced pandemic control in Kyoto City, Japan.

## Materials and methods

### Study setting and study design

This was a population-based, retrospective observational cohort study conducted in Kyoto City, Japan. Kyoto City is the capital of Kyoto Prefecture and one of 20 designated cities in Japan, with an estimated population of 1,454,000 people, including approximately 410,000 (28%) people aged over 65 years. Under the Infectious Disease Control Law, all confirmed cases of COVID-19 were registered in a national database, along with clinical information during the study period [9]. In this study, we used a local database in Kyoto City, which included all residents who were diagnosed with COVID-19. The database comprised completely anonymized data, including age, sex, testing samples, testing methods, testing facilities, and symptoms from the beginning of January 2020 to the end of June 2022, spanning a total of 30 months.

### Definitions

#### COVID-19 cases and diagnostic modalities

COVID-19 cases were diagnosed with the following:

- 1) the detection of the pathogen by isolation and identification or the genes by direct nucleic acid amplification test (NAAT) from sputum, tracheal aspirate, alveolar lavage fluid, pharyngeal swab, nasal aspirate, nasal swab, nasopharyngeal swab, stool, saliva, autopsy material specimens and other materials suitable for testing methods.
- 2) the detection of pathogen antigens by lateral flow device (LFD) from nasal swabs or nasopharyngeal swabs.
- 3) the detection of infection in antigen qualitative tests using automated immunoassays (AQT) from nasal swabs, nasopharyngeal swabs, or saliva.

In a few cases, a “clinical diagnosis” was made if the patients were in close contact with COVID-19 patients and had flu-like symptoms [10]. The list of approved testing methods and testing kits is attached in the supplement table and as published on the website of the Ministry of Health, Labour and Welfare, Japan [7].

### Symptomatic presentations

In the database, the patients’ symptomatic conditions at the time of testing were reported as fever, fatigue, cough or runny nose, difficulty breathing, nausea or vomiting, diarrhea, disturbance of consciousness, dysuria, and others, unless they were reported as asymptomatic.

### Testing facility types

The testing sites that collected specimens during the study period were categorized into seven categories: public health centre in Kyoto City, public facilities except for the public health centre (other public facilities), hospitals with beds reserved for people with SARS-CoV-2 infections (COVID hospitals), hospitals that did not have beds reserved for people with SARS-CoV-2 infections (non-COVID hospitals), general practitioners, elderly care facilities, and other facilities engaged in testing (others).

### Infection control policy in Kyoto City during the study period

In Kyoto City, COVID-19 tests were performed in medical institutions for symptomatic patients. In addition, the local health care centre in Kyoto City actively traced and tested close contacts to identify asymptomatic cases. Initially, during the pandemic, administrative COVID-19 testing was conducted for all cases among close contacts at the public health centre. However, starting in August 2021, when the Delta variant started to disseminate, administrative COVID-19 testing for asymptomatic cases was limited to close contacts within households.

### Statistical analysis

We computed the distribution of symptomatic presentation (symptomatic, asymptomatic) among confirmed cases over a 30-month period. We applied the chi-square test to assess the statistical significance of the distribution of test methods (NAAT, LFD, AQT, and clinical diagnosis) and testing locations throughout the course of the pandemic.

All analyses were performed using the Statistical Analysis System (SAS) statistical software package, version 9.1.3. (SAS Institute Inc., Cary, NC, USA). Two-tailed p values less than 0.05 were considered statistically significant.

## Results

### Trend of COVID-19 cases and symptomatic presentation

**Fig. 1:**
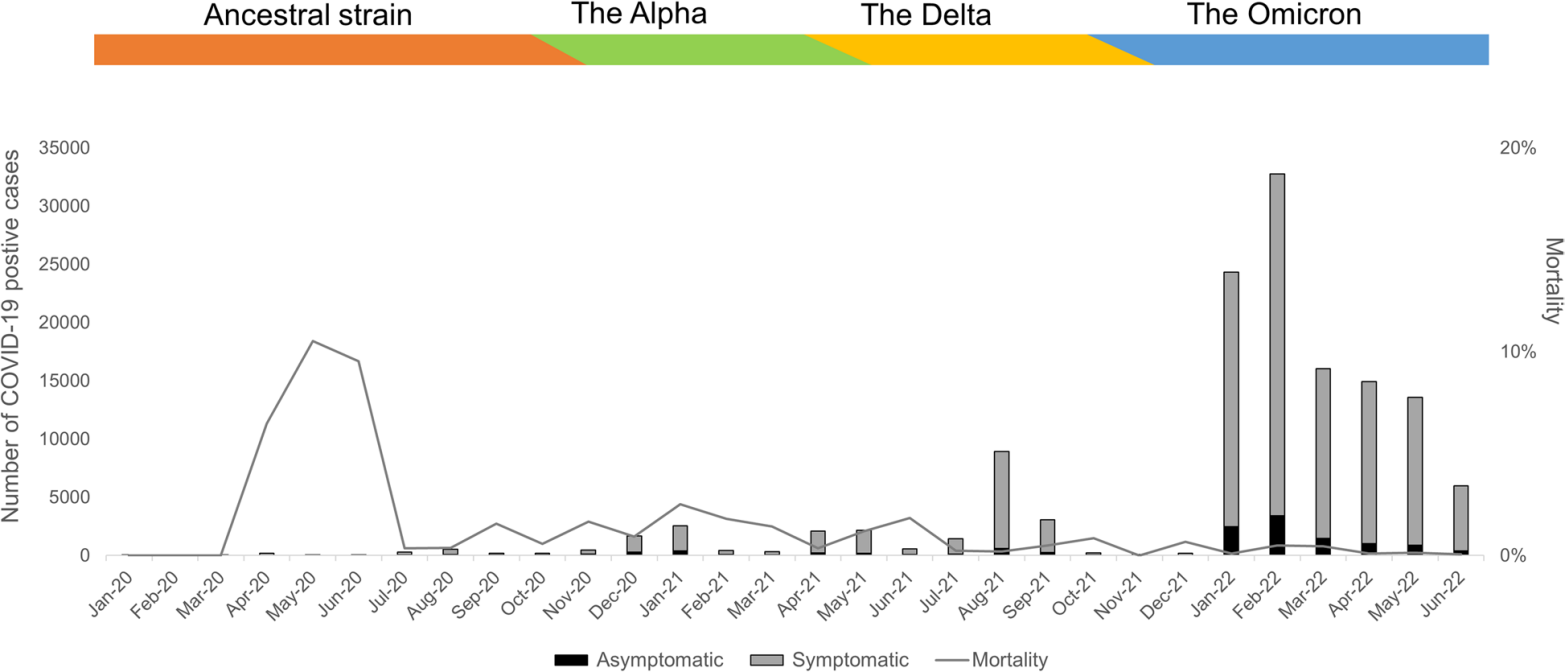
Trend in Testing Volume and Symptomatic Presentation of Confirmed Cases in Kyoto City. Histogram of the total number of SARS-CoV-2 tests and symptomatic presentation of patients examined and reported on the date of testing. The number of tests per month with patients’ symptomatic presentations reported to Kyoto City is shown in each column (*n* = 133,115) from January 2020 to June 2022. 80.8% of confirmed cases were diagnosed in 2022 during the Omicron surge, and the highest numbers were identified in February 2022 (24.6% of all tests), while only 2.7% (3,603) were identified in 2020. On the graph, the major variant strains that were prevalent in Japan and the mortality in Kyoto City are also indicated for reference

During the study period from January 1, 2020, to June 31, 2022, 133,115 subjects were reported to have COVID-19 infections in Kyoto City. The trend of the testing showed that 80.8% of confirmed cases were diagnosed in 2022 during the Omicron surge, and the highest numbers of both symptomatic (29,360) and asymptomatic (3,403) cases were identified in February 2022 (24.6% of all tests), while only 2.7% (3,603) were identified in 2020 (Fig. 1). The mortality due to COVID-19 during the study period was 0.4%. The trend of mortality in Kyoto City and the status of the predominant variant strains in Japan during the study period were illustrated in Fig. 1. (https://www.mhlw.go.jp/stf/covid-19/kokunainohasseijoukyou_00006.html). During the study period, a total of 9.1% of patients (12,149) were asymptomatic, while nine out of ten showed symptoms (120,966). The proportions of asymptomatic cases were higher in 2020 (16.4%, peaking at 22.5% in November 2020) than in the total asymptomatic proportion of 9.1% (8.9% and 9.0% in 2022 and 2021, respectively). The distribution of asymptomatic and symptomatic cases significantly differed over time (*p* < 0.0001).

### Trend in the type of testing

The type of testing was also analyzed. (supplement Fig. 1A) All testing increased until the peak in February 2022 (32,763 cases per month), when the Omicron variant was the predominant strain. For an extended period, NAAT was the predominant diagnostic method for SARS-CoV-2 diagnosis in Japan, accounting for 68.9% of all testing. However, the ratio of LFD use gradually increased from the end of 2020 and rapidly increased in 2022 (supplement Fig. 1B). The total LFDs tested in 2022 was a 351% increase from the LFDs tested in 2021. As such, the distribution of testing methods significantly differed over the 30 months (*p* < 0.0001).

### Trend in testing facilities

**Fig. 2:**
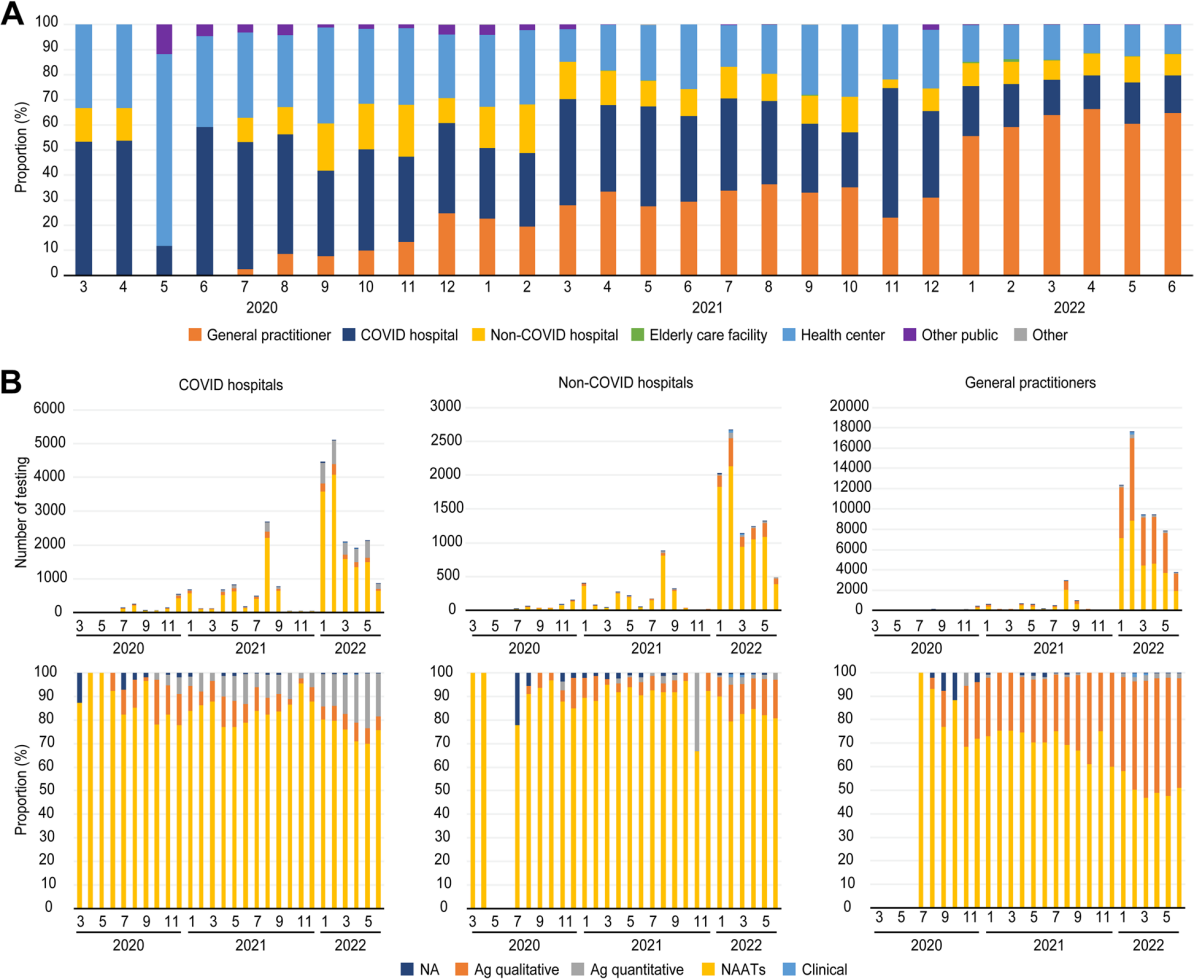
Trend in total testing facilities and type of testing used in three major facilities in Kyoto City **A** The proportion of functional health facilities that collected SARS-CoV-2 specimens in Kyoto City. **B** Trend and proportion of SARS-CoV-2 testing methods at three facilities in Kyoto City. **A** Histograms of the total proportion of functional health facilities that collected SARS-CoV-2 specimens are shown in each column from January 2020 to June 2022. A total of 811 facilities that collected specimens each month were reported and integrated into seven main categories: public health centre, other public facilities, COVID hospitals, non-COVID hospitals, general practitioners, elder care facilities, and others. The proportion of diagnoses by general practitioners at outpatient primary health clinics gradually increased and reversed in July 2021, becoming the leading facility type to provide SARS-CoV-2 testing. **B** Histograms of the total number of tests and the type of testing for SARS-CoV-2 in three main facilities from January 2020 to June 2022 are shown: COVID-19 hospitals (*n* = 24,500), non-COVID-19 hospitals (*n* = 11,774), and general practitioners (*n* = 67,512)

The number of functional testing facilities during the study period was 811. At the beginning of the pandemic, the testing capability was limited in certain facilities, such as the public health centre and COVID-19 hospitals. Although the hospitals that performed SARS-CoV-2 treatment (COVID hospitals) tested the most people until June 2021, the proportion of diagnoses by general practitioners at outpatient primary health clinics gradually increased and reversed in July 2021, becoming the leading facility type to provide SARS-CoV-2 testing (Fig. 2). With the upward trend in testing by general practitioners, 10.4% of patients were diagnosed in hospitals that did not perform SARS-CoV-2 care (non-COVID hospitals), and the ratio did not show significant fluctuation compared to others. In this manner, the distribution of testing facilities significantly differed over the 30 months (*p* < 0.0001).

### Testing methods by health care facilities

Among all the subjects who reported being tested by general practitioners (67,512), 44.4% (29,953) of the testing was performed with LFDs (Fig. 2B), while LFDs accounted for 26.3% of the total testing methods of all facilities (supplement Fig. 1B). Our research also showed that NAATs were widely performed in all three types of facilities; the physicians at non-COVID hospitals used the highest ratio of NAATs (85.3% of all tests at non-COVID hospitals) compared to those at other facilities (78.6% and 52.8% in COVID hospitals and among general practitioners, respectively).

### Testing specimens by health care facilities

Among the 132,986 reported cases, saliva was the most commonly used specimen (56,794, 42.8% of the total), and the number increased mainly in 2022. On the other hand, nasopharyngeal swab specimen use was reduced by 25.7% when comparing the ratio between 2020 and 2022 (50.9% to 37.8%) (supplement Fig. 2).

### Type of specimen and testing method

**Fig. 3:**
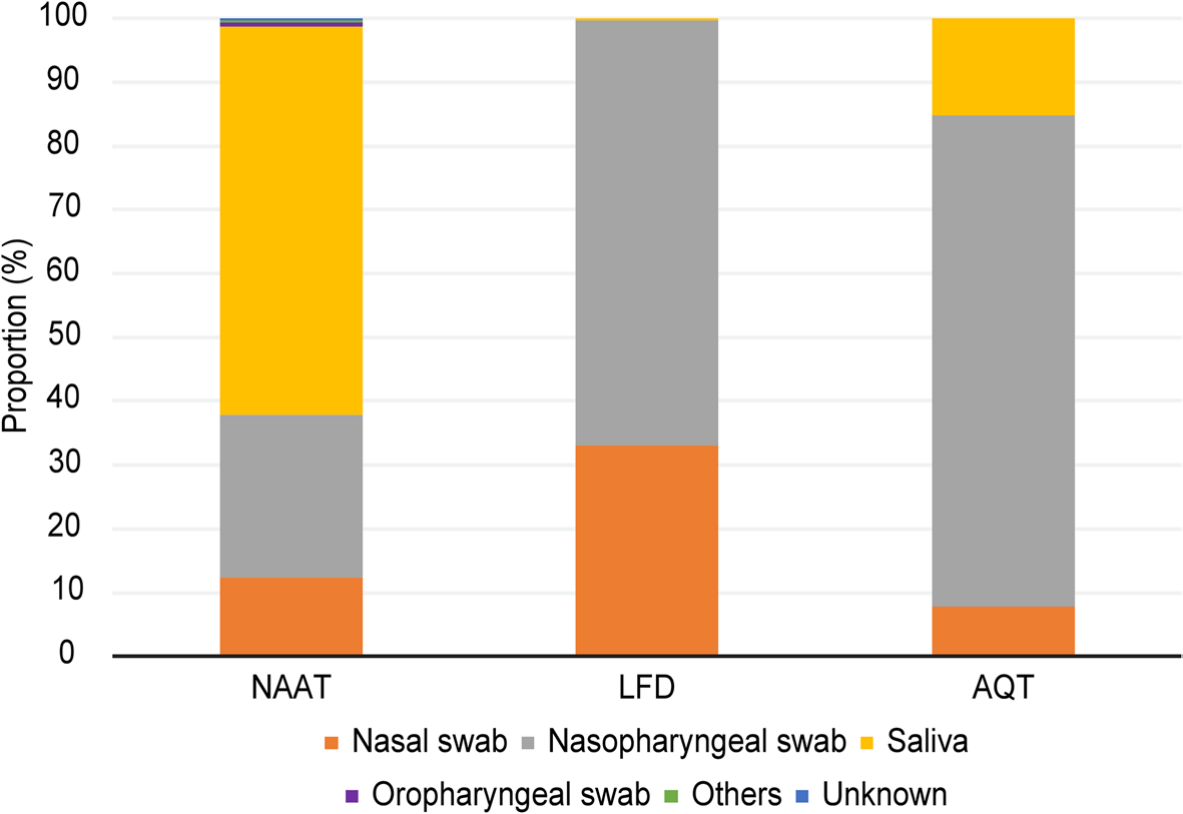
Types of Specimens in NAAT and Antigen Tests. The total numbers of specimens were 91,176 in NAAT, 34,529 in lateral flow device (LFD) testing, and 5,257 in antigen qualitative tests using automated immunoassays (AQT) (*n* = 130,962). While saliva samples were most commonly utilized for NAAT (61%), nasopharyngeal swab samples were predominantly used for LFD and AQT (67% and 77%, respectively)

A total of 130,962 subjects’ specimens for each type of testing were recorded. While saliva samples were most commonly utilized for NAAT (61%), nasopharyngeal swab samples were predominantly used for LFD and AQT (67% and 77%, respectively) (Fig. 3).

## Discussion

This is the first population-based study analyzing the dynamic shift in testing utilization from the beginning of the pandemic to the most recent Omicron surge in 2022 in Japan.

In the early stages of the pandemic, the capacity of SARS-CoV-2 testing was limited due to delays in the introduction of molecular diagnostic systems for pathogens in clinical laboratories in Japan. According to Mathieu et al., the number of tests per 1,000 people in Japan was almost 1/10 to 1/50 that performed in the United States and other developed countries in 2020 [8, 10]. At that time, the public health centre or COVID-19 hospitals were the places where most cases were tested, as indicated in this study. However, with the widespread adoption of LFDs and AQTs, the landscape of testing and diagnosis dramatically shifted. This study also revealed that revisions in the scope of active epidemiological investigations (close contact investigation) also influenced the changes in the proportion of asymptomatic cases and the testing facilities. For example, the decrease in the proportion of testing conducted at the public health centre (where administrative close contact investigations were mainly performed) resulted in changes in the proportion of asymptomatic cases. It is certain that the shift in policy towards not actively testing asymptomatic individuals has made it more difficult to determine the transmission/infection rates for each variant in each phase and may mask disease burden. However, there will be a continued need to consider the balance between the appropriate use of testing, resource conservation, and the impact asymptomatic individuals may have on public health from here on out [11].

According to this study, primary outpatient clinics of general practitioners and non-COVID hospitals gradually played a more important role, especially in the Omicron surge. In addition, tests using methods of antigen detection (LFDs and AQTs) increased dramatically in 2022. While increased testing capacity in outpatient primary care facilities using antigen testing seemed to be beneficial for evaluating infection control measures and understanding the pandemic, the lower sensitivity of antigen tests, which is even worse depending on the presence of mutations in viral genomes, has a risk of missing truly infected cases [12–16]. However, even NAATs still miss a certain number of cases, and it has been recently revealed that the accuracy of antigen tests, depending on the specimen and target, is not necessarily inferior to NAATs [17–21]. These characteristics of the testing, such as incompleteness and detection limits, should be widely understood, and a system that can reliably diagnose the disease by selecting patients at high risk to implement rapid and efficient infection control measures is needed.[22] At present, NAAT is still the gold standard test with higher sensitivity for viral infection in clinical settings and we believe the parallel expansion and efficient use of antigen testing is helpful in establishing a more comprehensive and risk-reducing strategy during a pandemic [14, 23–26].

Our research showed that nasopharyngeal swabs were the most common specimens used for antigen testing. The advantages of antigen testing are that it is inexpensive and a simple and rapid testing method, especially when performed as point-of-care testing. Since nasopharyngeal swab samples cannot be self-collected, it may be desirable to develop POCT options with high sensitivity using self-collected samples, including saliva. Saliva specimens were frequently utilized for SARS-CoV-2 testing due to the ease of self-collection and the reduced burden on patients compared to nasopharyngeal swabs [27–29]. Although saliva requires preprocessing due to individual variability, the challenges associated with using saliva for testing are relatively few. Nevertheless, there is a need to explore simpler and more accurate testing methods, including those utilizing saliva. This approach would allow the advantages of POCT to be fully realized [28]. In addition, as there are limitations of sensitivity in some testing methods, particularly along asymptomatic patients, retesting over time should be asserted to reduce omitted cases [20]. From the perspective of public health advantages, the Food and Drug Administration (FDA) has recommended repeat testing after a negative-result individual although they present COVID-19 symptoms; for example, at least three tests over five days (with 48 h between tests) for asymptomatic individuals are recommended [30]. In addition, repeat testing was shown to be effective for improving contact tracing in some settings. It would be necessary to widely disseminate knowledge about the differentiation of testing methods based on the pathogenesis of infectious diseases and testing purposes [31, 32].

This study, being a retrospective observational analysis, requires acknowledgment of certain inherent limitations. First, all COVID-19 positive cases were mandated by law to be registered, ensuring no omissions in the reported case numbers. However, there was a possibility of missing data or errors due to instances where public health officials manually entered information. Furthermore, this analysis, focusing exclusively on COVID-19-positive cases, has limitations due to variations in the sensitivity and specificity of the testing, depending on the testing kits, NAAT reagents used, and the combination of the target population. Calculating the positivity rate based on the number of tests conducted for each testing method should allow for an examination of the impact of changes in testing methods on the epidemiological investigation of COVID-19. Particularly, the anticipated increase in the frequency of rapid diagnostic tests may lead to the emergence of undiagnosed cases. Further research through modeling studies is necessary to assess the implications of changes in testing methods on public health.

This study revealed that COVID-19 testing underwent statistically significant changes during the pandemic in Kyoto City. By comprehending and documenting the evolution of testing methods and testing locations, it is anticipated that this will contribute to the establishment of an even more efficient testing infrastructure. In this pandemic, it became evident that there were challenges not only in establishing testing methods but also in all aspects surrounding testing, including logistics, accuracy management, and human resources [2]. With pathogens, once the genome sequence is known, primer design is possible. In the event of a pandemic, the foremost priority is to expedite widespread testing for maximum efficiency. Lessons for future pandemics in terms of testing include maintaining testing technology during non-pandemic periods and stabilizing logistics. Both testing industries and academia are required to take the lead in establishing a rapid and practical conduit for testing systems as a part of emergency preparedness for future pandemics.

### Supplementary Information

**Additional file 1.** 

** Additional file 2.** 

** Additional file 3.**

## Data Availability

The data that support the findings of this study are available from the corresponding author upon reasonable request.
