# Supplementary material for: A population-based study of the trend in SARS-CoV-2 diagnostic modalities from the beginning of the pandemic to the Omicron surge in Kyoto City, Kyoto, Japan
Source: BMC Public Health. 2023 Dec 21;23:2551. doi: 10.1186/s12889-023-17498-3 (PMC10734122; doi:10.1186/s12889-023-17498-3)
Supplement: Supplementary file 1 — Additional file 1. [file 12889_2023_17498_MOESM1_ESM.docx]

Supplement table

List of approved testing methods and kits for COVID-19 during study period

**1. Nucleic Acid Amplification Method**

| No. | Product name | Marketing Authorization Holder | Type | Date of Authorization |
| --- | --- | --- | --- | --- |
| 1 | 2019-nCoV Fluorescence Detection Real-time RT-PCR Kit | Sysmex Corporation | Nucleic acid （RT-PCR） | March 27, 2020 |
| 2 | Loopamp Novel Coronavirus 2019 (SARS-CoV-2) Detection Kit | Eiken Chemical Co., Ltd. | Nucleic acid （LAMP） | March 31, 2020 |
| 3 | cobas SARS-CoV-2 | Roche Diagnostics K.K. | Nucleic acid （RT-PCR） | April 7, 2020 |
| 4 | TaqPath Real Time PCR Reagent Kit for SARS-CoV-2 | Life Technologies Japan Ltd. | Nucleic acid （RT-PCR） | April 20, 2020 |
| 5 | Xpert Xpress SARS-CoV-2 ‘Cepheid’ | Beckman Coulter, Inc. | Nucleic acid （RT-PCR） | May 8, 2020 |
| 6 | MEBRIGHT SARS-CoV-2 Kit | MEDICAL＆BIOLOGICAL LABORATORIES CO., LTD. | Nucleic acid （RT-PCR） | May 21, 2020 |
| 7 | FilmArray Respiratory Panel 2.1 | bioMérieux Japan Ltd. | Nucleic acid （RT-PCR） | June 2, 2020 |
| 8 | GENECUBE SARS-CoV-2 | TOYOBO CO., LTD. | Nucleic acid （RT-PCR） | July 2, 2020 |
| 9 | TRCReady SARS-CoV-2 | TOSOH CORPORATION | Nucleic acid （TRC） | July 31, 2020 |
| 10 | SmartAmp SARS-CoV-2 | K.K. DNAFORM | Nucleic acid (SmartAmp) | August 17, 2020 |
| 11 | Aptima SARS-CoV-2 | Hologic Japan, Inc. | Nucleic acid (TMA) | August 18, 2020 |
| 12 | Ampdirect 2019-nCoV Detection Kit | SHIMADZU CORPORATION | Nucleic acid （RT-PCR） | September 8, 2020 |
| 13 | i-densy Pack SARS-CoV-2 | ARKRAY Factory, Inc. | Nucleic acid （RT-PCR） | September 8, 2020 |
| 14 | ID NOW COVID-19 | Abbott Diagnostics Medical Co., Ltd | Nucleic acid (Isothermal Amplification) | October 20, 2020 |
| 15 | GENECUBE HQ SARS-CoV-2 | TOYOBO CO., LTD. | Nucleic acid （RT-PCR） | October 23, 2020 |
| 16 | SGNP nCoV/Flu PCR Detection Kit | SUDx-Biotec Corporation | Nucleic acid （RT-PCR） | October 23, 2020 |
| 17 | Takara SARS-CoV-2 Direct PCR detection kit | Takara Bio Inc. | Nucleic acid （RT-PCR） | October 27, 2020 |
| 18 | cobas SARS-CoV-2 & Flu A/B | Roche Diagnostics K.K. | Nucleic acid （RT-PCR） | November 13, 2020 |
| 19 | Illumina COVIDSeq Test | Illumina K.K. | Next Generation Sequencing | December 8, 2020 |
| 20 | TaqPath SARS-CoV-2 Real Time PCR Reagent Kit HT | Life Technologies Japan Ltd. | Nucleic acid （RT-PCR） | December 17, 2020 |
| 21 | TRCReady SARS-CoV-2 i | TOSOH CORPORATION | Nucleic acid （TRC） | January 19, 2021 |
| 22 | Simprova SARS-CoV-2 Detection Kit | Eiken Chemical Co., Ltd. | Nucleic acid （LAMP） | February 15, 2021 |
| 23 | Takara SARS-CoV-2 & Flu Direct PCR detection kit | Takara Bio Inc. | Nucleic acid （RT-PCR） | February 15, 2021 |
| 24 | Smart Gene SARS-CoV-2 | MIZUHO MEDY Co.,Ltd | Nucleic acid （RT-PCR） | February 18, 2021 |
| 25 | Alinity m system SARS-CoV-2 | Abbott Japan LLC | Nucleic acid （RT-PCR） | February 24, 2021 |
| 26 | cobas Liat SARS-CoV-2 & Flu A/B | Roche Diagnostics K.K. | Nucleic acid （RT-PCR） | March 12, 2021 |
| 27 | Xpert Xpress SARS-CoV-2/Flu‘Cepheid’ | Beckman Coulter, Inc. | Nucleic acid （RT-PCR） | April 14, 2021 |
| 28 | DetectAmp SARS-CoV-2 RT-PCR Kit | Sysmex Corporation | Nucleic acid （RT-PCR） | April 14, 2021 |
| 29 | DELBio Dagane G1 SARS-CoV-2 Detection Kit | DELTA ELECTRONICS (JAPAN), INC. | Nucleic acid （RT-PCR） | May 27, 2021 |
| 30 | SGNP nCoV PCR detection kit | SUDx-Biotec Corporation | Nucleic acid （RT-PCR） | May 27, 2021 |
| 31 | Swiftgene SARS-CoV-2 KAINOS | KAINOS Laboratories, Inc. | Nucleic acid (Isothermal Amplification) | May 31, 2021 |
| 32 | TRexGene SARS-CoV-2 Detection kit | TOYOBO CO., LTD. | Nucleic acid （RT-PCR） | June 3, 2021 |
| 33 | ELITe MGB SARS-CoV-2 PCR Detection Kit | Precision System Science Co., Ltd. | Nucleic acid （RT-PCR） | June 21, 2021 |
| 34 | KANEKA Direct RT-PCR kit SARS-CoV-2 | KANEKA CORPORATION | Nucleic acid （RT-PCR） | June 25, 2021 |
| 35 | FTD SARS-CoV2 Kit | Siemens Healthcare Diagnostics K.K. | Nucleic acid （RT-PCR） | November 17, 2021 |
| 36 | μTASWako SARS-CoV-2 | FUJIFILM Wako Pure Chemical Corporation | Nucleic acid （RT-PCR） | November 30, 2021 |
| 37 | J-Bio Rapid PCR Kit SARS-CoV-2 | Japan Biotechno Pharma Co.,Ltd | Nucleic acid （RT-PCR） | December 23, 2021 |
| 38 | BD SARS-CoV-2/Flu for BD MAX System | Becton, Dickinson and Company | Nucleic acid （RT-PCR） | January 31, 2022 |
| 39 | GeneSoC SARS-CoV-2 N2 Detection Kit | KYORIN Pharmaceutical Co., Ltd. | Nucleic acid （RT-PCR） | March 2, 2022 |
| 40 | LumiraDx SARS-CoV-2 RNA STAR Complete | LumiraDx Japan Co., Ltd. | Nucleic acid （RT-PCR） | March 31, 2022 |
| 41 | GENECUBE HQ SARS-CoV-2/RSV | TOYOBO CO., LTD. | Nucleic Acid （RT-PCR） | April 6, 2022 |
| 42 | GSARS-CoV-2 RNA detection kit LAMPdirect | CANON MEDICAL SYSTEMS CORPORATION | Nucleic Acid (LAMP) | April 8, 2022 |

**2. Antigen Test**

| No. | Product name | Marketing Authorization Holder | Type | Date of Authorization |
| --- | --- | --- | --- | --- |
| 1 | ESPLINE SARS-CoV-2 | Fujirebio Inc. | Antigen (simple kit) | May 13, 2020 |
| 2 | Lumipulse G SARS-CoV-2 Ag | Fujirebio Inc. | Antigen (quantitative test) | June 19, 2020 |
| 3 | QuickNavi-COVID19 Ag | Denka Co., Ltd. | Antigen (simple kit) | August 11, 2020 |
| 4 | ImunoAce SARS-CoV-2 Capilia SARS-CoV-2 | TAUNS LABORATORIES, INC. | Antigen (simple kit) | October 13, 2020 |
| 5 | Lumipulse Presto SARS-CoV-2 Ag | Fujirebio Inc. | Antigen (quantitative test) | October 16, 2020 |
| 6 | HISCL SARS-CoV-2 Ag Assay Kit | Sysmex Corporation | Antigen (quantitative test) | November 10, 2020 |
| 7 | SARS-CoV-2 Antigen Rapid Test Kit Rapiim SARS-CoV-2-N PRT-C2N01A | CANON MEDICAL SYSTEMS CORPORATION | Antigen (qualitative test) | December 8, 2020 |
| 8 | LumiraDx SARS-CoV-2 Ag Test Strip | LumiraDx Japan Co., Ltd. | Antigen (qualitative test) | January 19, 2021 |
| 9 | Panbio^TM^ COVID-19 Ag Rapid Test Device(NASOPHARYNGEAL)/ Panbio^TM^ COVID-19 Ag Rapid Test Device(NASAL) | Abbott Diagnostics Medical Co., Ltd. | Antigen (simple kit) | January 22, 2021 |
| 10 | BD Veritor System for Rapid Detection of SARS-CoV-2 | Becton, Dickinson and Company | Antigen (qualitative test) | January 26, 2021 |
| 11 | PRORAST SARS-CoV-2 Ag ADTest SARS-CoV-2 | ADTEC Corporation／LSI Medience Corporation | Antigen (simple kit) | January 29, 2021 |
| 12 | SARS-CoV-2 Rapid Antigen Test | Roche Diagnostics K.K. | Antigen (simple kit) | February 9, 2021 |
| 13 | FUJI DRI-CHEM IMMUNO AG HANDY COVID-19 Ag | FUJIFILM Corporation | Antigen (simple kit) | February 15, 2021 |
| 14 | Sofia SARS Antigen FIA | Quidel Corporation | Antigen (qualitative test) | February 18, 2021 |
| 15 | Quick Chaser Auto SARS-CoV-2 | MIZUHO MEDY Co.,Ltd | Antigen (qualitative test) | March 2, 2021 |
| 16 | FUJI DRI-CHEM IMMUNO AG CARTRIDGE COVID-19 Ag | MIZUHO MEDY Co.,Ltd | Antigen (qualitative test) | March 2, 2021 |
| 17 | ALSONIC COVID-19 Ag | Alfresa Pharma Corporation | Antigen (simple kit) | March 12, 2021 |
| 18 | KBM LineCheck nCoV (Stick Type) | KOHJIN BIO CO., LTD. | Antigen (simple kit) | March 17, 2021 |
| 19 | Elecsys SARS-CoV-2 Antigen | Roche Diagnostics K.K. | Antigen (quantitative test) | March 17, 2021 |
| 20 | COVID-19 and Influenza A+B Antigen Combo Test (NichireiBio) | Nichirei Biosciences Inc. | Antigen (simple kit) | April 14, 2021 |
| 21 | ImmunoArrow SARS-CoV-2 | TOYOBO CO., LTD. | Antigen (simple kit) | May 12, 2021 |
| 22 | VITROS SARS-CoV-2 Antigen | ORTHO-CLINICAL DIAGNOSTICS K.K. | Antigen (quantitative test) | May 12, 2021 |
| 23 | Check MR-COV19 dotest COV19 | ROHTO Pharmaceutical Co., Ltd. | Antigen (simple kit) | May 27, 2021 October 11, 2021 |
| 24 | RapidTesta SARS-CoV-2 | SEKISUI MEDICAL CO., LTD. | Antigen (simple kit) | June 9, 2021 |
| 25 | AFIAS COVID-19 Ag Test Cartridge | Prime fine Co., Ltd. (succeeded from TOKYO BOEKI MEDISYS INC.) | Antigen (qualitative test) | June 16, 2021 |
| 26 | QuickNavi-Flu+COVID19 Ag | Denka Co., Ltd. | Antigen (simple kit) | June 16, 2021 |
| 27 | STANDARD^TM^ Q COVID-19 Ag Test | MALCOM COMPANY LIMITED | Antigen (simple kit) | August 13, 2021 |
| 28 | ESPLINE SARS-CoV-2&FLU A+B | FUJIREBIO INC. | Antigen (simple kit) | August 13, 2021 |
| 29 | Quampas COVID-19 Antigen Test Kit | Cellspect Co.,Ltd. | Antigen (simple kit) | September 14, 2021 |
| 30 | Immunofine SARS-COV-2 | Nichirei Biosciences Inc. | Antigen (simple kit) | September 14, 2021 |
| 31 | CL AIA-PACK SARS-CoV-2-Ag | TOSOH CORPORATION | Antigen (quantitative test) | September 14, 2021 |
| 32 | ImunoAce SARS-CoV-2 Ⅱ/ Capilia SARS-CoV-2 Ⅱ | TAUNS Laboratories,Inc. | Antigen (simple kit) | November 8, 2021 |
| 33 | KBM LineCheck nCoV/Flu | KOHJIN BIO CO., LTD. | Antigen (simple kit) | November 8, 2021 |
| 34 | Quick Chaser SARS-CoV-2/Flu | MIZUHO MEDY Co.,Ltd | Antigen (simple kit) | November 17, 2021 |
| 35 | Quick Chaser SARS-CoV-2/Flu A,B | MIZUHO MEDY Co.,Ltd | Antigen (simple kit) | November 17, 2021 |
| 36 | Accuraseed SARS-CoV-2Ag | FUJIFILM Wako Pure Chemical Corporation | Antigen (simple kit) | November 30, 2021 |
| 37 | ADTest SARS-CoV-2/Flu | ADTEC CO.,LTD. | Antigen (simple kit) | February 8, 2022 |
| 38 | Primal Screen SARS-CoV-2/Flu | ADTEC CO.,LTD. | Antigen (simple kit) | February 8, 2022 |
| 39 | ImunoAce SARS-CoV-2/Flu / Capilia SARS-CoV-2 /Flu | TAUNS Laboratories,Inc. | Antigen (simple kit) | February 8, 2022 |
| 40 | GLINE-2019-nCoV Ag Kit | MEDICAL & BIOLOGICAL LABORATORIES CO., LTD. | Antigen (simple kit) | February 10, 2022 |
| 41 | Exdia EK Test COVID-19 Ag | EIKEN CHEMICAL CO.,LTD. | Antigen (qualitative test) | February 10, 2022 |
| 42 | CLINITEST Rapid COVID-19 Antigen Self-Test | Siemens Healthcare Diagnostics K.K. | Antigen (simple kit) | February 16, 2022 |
| 43 | ADTest SARS-CoV-2 NEO | ADTEC CO.,LTD. | Antigen (simple kit) | February 24, 2022 |
| 44 | COVID-19 Antigen Test (NichireiBio) | NICHIREI BIOSCIENCES INC. | Antigen (simple kit) | March 4, 2022 |
| 45 | ImunoAce SARS-CoV-2 Saliva / Capilia SARS-CoV-2  Saliva | TAUNS Laboratories,Inc. | Antigen (simple kit) | March 17, 2022 |
| 46 | HEALGEN Rapid COVID-19 Antigen Self-Test | Takara Bio Inc. | Antigen (simple kit) | March 17, 2022 |
| 47 | LumiraDx SARS-CoV-2 & Flu A/B Test Strip | LumiraDx Japan Co., Ltd. | Antigen (qualitative test) | March 31, 2022 |
| 48 | Quick Chaser SARS-CoV-2 | MIZUHO MEDY Co.,Ltd | Antigen (simple kit) | April 19, 2022 |
| 49 | KANEKA Immunochromatography SARS-CoV-2 Ag | KANEKA CORPORATION | Antigen (simple kit) | May 25, 2022 |
| 50 | Inspecter Kowa SARS-CoV-2 | KOWA Co., Ltd. (succeeded from MEDICAL＆BIOLOGICAL LABORATORIES CO., LTD.) | Antigen (simple kit) | June 1, 2022 |
| 51 | ESPLINE SARS-CoV-2 N | Fujirebio Inc. | Antigen (simple kit) | June 7, 2022 |
