# Supplementary figures and images for: A population-based study of the trend in SARS-CoV-2 diagnostic modalities from the beginning of the pandemic to the Omicron surge in Kyoto City, Kyoto, Japan
Source: BMC Public Health. 2023 Dec 21;23:2551. doi: 10.1186/s12889-023-17498-3 (PMC10734122; doi:10.1186/s12889-023-17498-3)

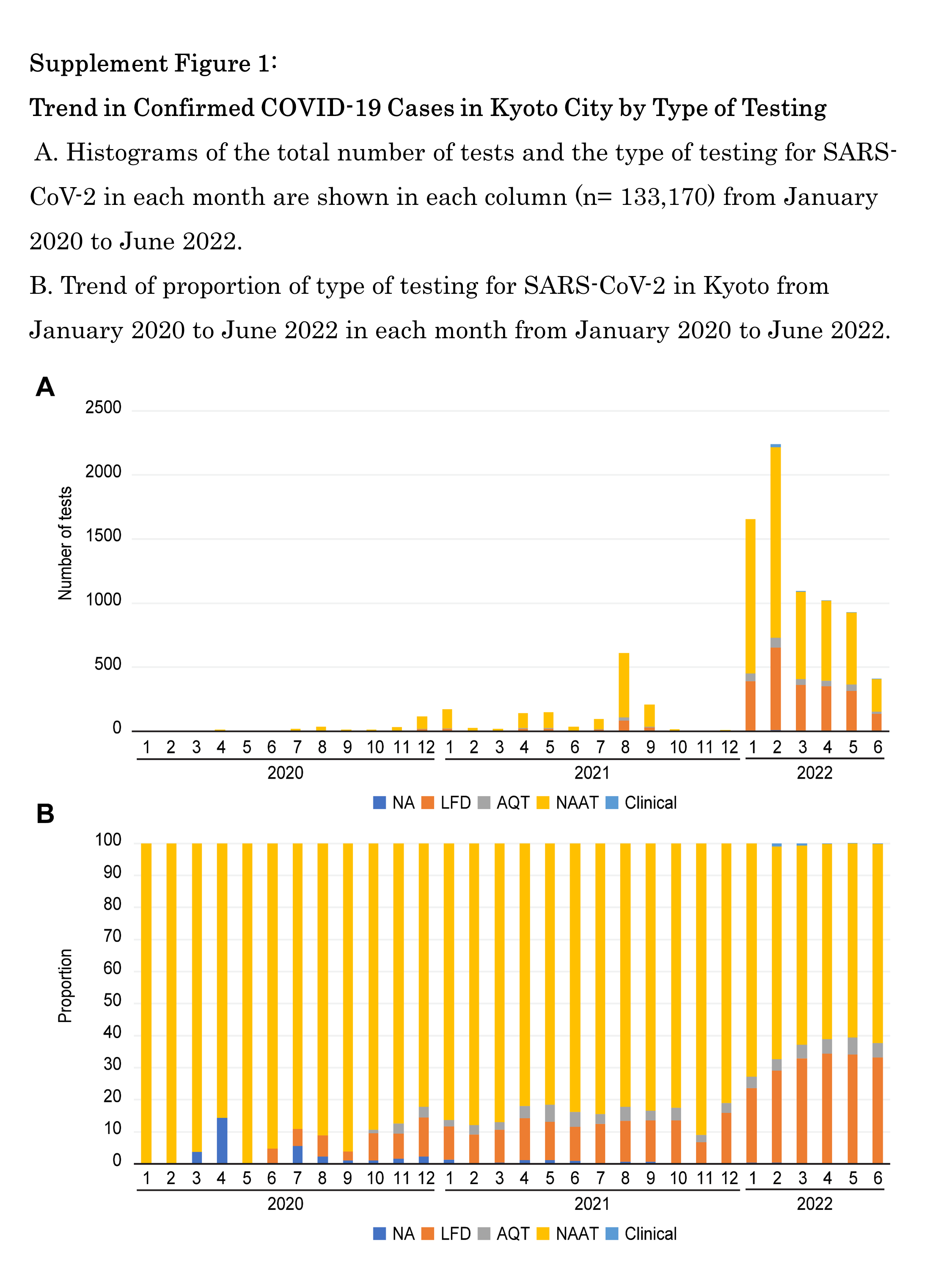

Supplement: Supplementary file 2 — Additional file 2. [file 12889_2023_17498_MOESM2_ESM.tif]

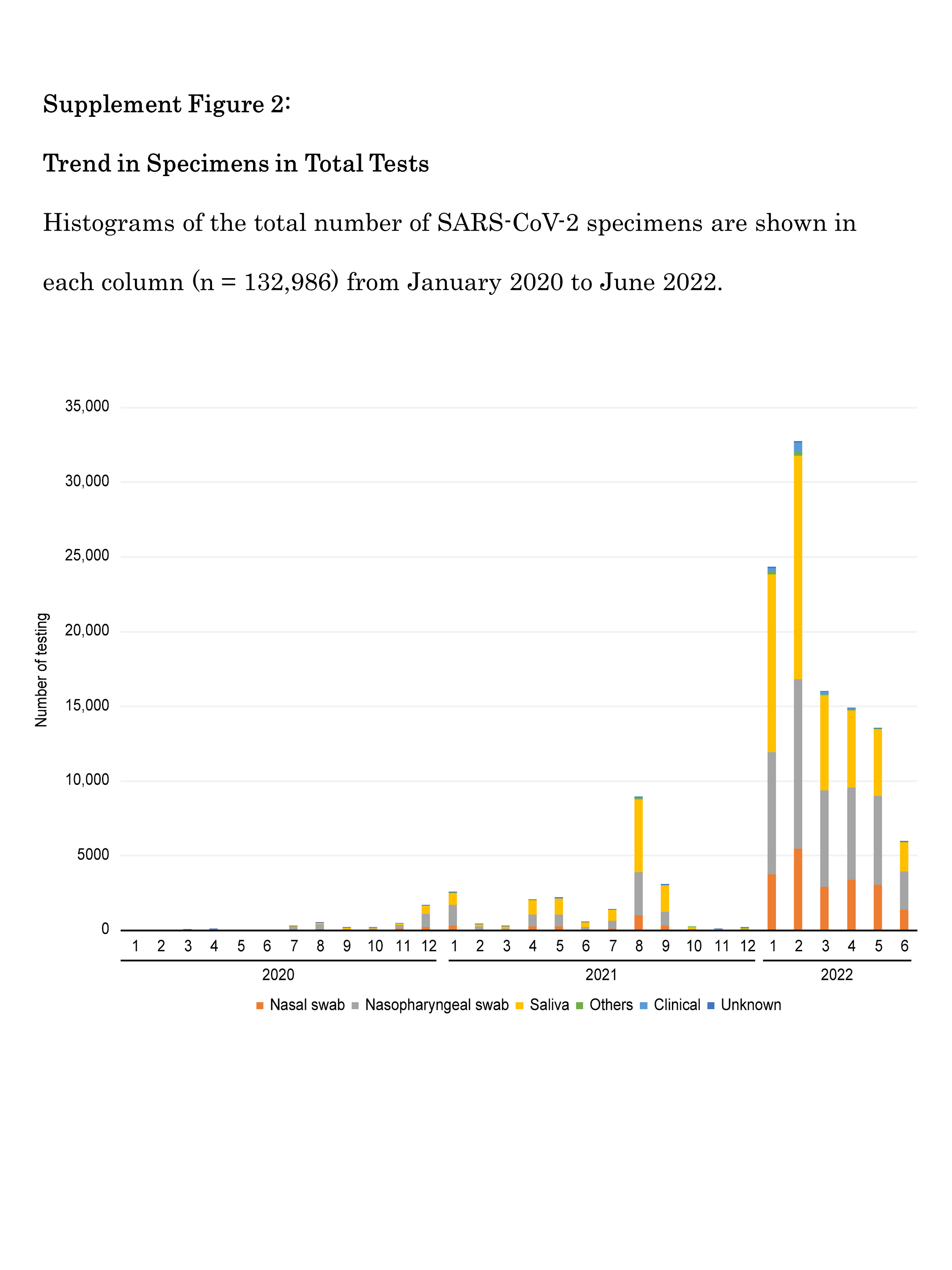

Supplement: Supplementary file 3 — Additional file 3. [file 12889_2023_17498_MOESM3_ESM.tif]
